# Stability of Inhaled Ciprofloxacin-Loaded Poly(2-ethyl-2-oxazoline) Nanoparticle Dry Powder Inhaler Formulation in High Stressed Conditions

**DOI:** 10.3390/ph15101223

**Published:** 2022-10-02

**Authors:** Mohammad Zaidur Rahman Sabuj, Md Abdur Rashid, Tim R. Dargaville, Nazrul Islam

**Affiliations:** 1 Pharmacy Discipline, School of Clinical Sciences, Queensland University of Technology (QUT), Brisbane, QLD 4000, Australia; 2 Department of Pharmaceutics, College of Pharmacy, King Khalid University, Abha 62529, Aseer, Saudi Arabia; 3 Faculty of Science, School of Chemistry and Physics, Queensland University of Technology, Brisbane, QLD 4000, Australia; 4 Centre for Immunology and Infection Control (CIIC), Queensland University of Technology, Brisbane, QLD 4000, Australia

**Keywords:** ciprofloxacin, dry powder inhaler, inhalation formulation, nanoparticles, poly(2-ethyl-2-oxazoline), stability

## Abstract

In this study, the stability of ciprofloxacin (CIP)-loaded poly(2-ethyl-2-oxazoline) (PEtOx) nanoparticles (NPs) was investigated at normal and high stressed conditions. The blank NPs were used to understand the intrinsic physicochemical properties of the polymer NPs under these storage conditions. The formulated NPs were prepared by a coassembly reaction and dried by lyophilization. The powder NPs were stored at controlled room temperature (25 °C) with normal relative humidity (RH) (43%) and high temperature (40 °C) and RH (75%). The stored samples were analyzed by determining the particle sizes, morphology, solid-state properties, thermal behavior, drug-polymer interactions, and aerosol performances over six months. The chemical stability of the formulations was determined by X-ray diffraction, attenuated total refection-Fourier transform infrared (ATR-FTIR), and high-performance liquid chromatography (HPLC) over six months under both conditions. The particle size of the blank PEtOx NPs significantly (*p* < 0.05) increased from 195.4 nm to 202.7 nm after 3 months at 40 °C/75% RH due to the moisture absorption from high RH; however, no significant increase was observed afterward. On the other hand, the sizes of CIP-loaded PEtOx NPs significantly (*p* < 0.05) reduced from 200.2 nm to 126.3 nm after 6 months at 40 °C/75% RH. In addition, the scanning electron microscopy (SEM) images revealed that the surfaces of CIP-loaded PEtOx NPs become smoother after 3 months of storage due to the decay of surface drugs compared to the freshly prepared NPs. However, transmission electron microscopy (TEM) images could not provide much information on drug decay from the nanoparticle’s surfaces. The fine particle fraction (FPF) of CIP-loaded PEtOx NPs dropped significantly (*p* < 0.05) after three months at 25 °C/43% RH and 40 °C/75% RH conditions. The reduced FPF of CIP-loaded PEtOx NPs occurred due to the drug decay from the polymeric surface and blank PEtOx NPs due to the aggregations of the NPs at high temperatures and RH. Although the aerosolization properties of the prepared CIP-loaded PEtOx NPs were reduced, all formulations were chemically stable in the experimental conditions.

## 1. Introduction

Pulmonary drug delivery is one of the modern drug delivery systems for severe diseases, especially lung-related disorders. It demonstrates better therapeutic effects in low doses compared to currently available oral and intravenous medicines [1]. However, inhalation therapy always faces the challenges of retaining its stability and compromises its aerosol performances after a certain period. In most cases, the dry powder inhaler (DPI) formulation compromised its aerosolization properties under unfavourable temperatures and relative humidity (RH) [2]. DPI formulations usually produce extensive forces among the particles at high temperatures and RH, including capillary, van der Waals, stable bridging, and mechanical integration [3,4,5]. These forces among the particles usually result in aggregations and adversely affect the aerosolization properties of the formulations [6]. Thus, DPI formulations are usually prepared in combination with carriers to enhance the aerosolization performance of the formulations and protect the drug molecules in adverse conditions [7]. L-leucine and sugar molecules, including, mannitol, sucrose, lactose, and trehalose are the most favourable aerosol enhancers [8]. All these excipients provided stability to the formulated drug molecules to some extent [2]. However, most of these carriers usually absorb moisture at high RH and affect the stability of the formulations especially affecting the flow properties of the DPI formulations [9]. Additionally, the sugar molecules usually showed crystallization natures under unfavourable temperatures or RH and adversely affect the aerosol performance of the formulated compounds [10]. Thus, drug-loaded sugar molecules are usually stored in optimized conditions and needed frequent monitoring to ensure their stability [11,12]. For example, L-leucine-based inhaled podovirus phage (phage PEV20)-ciprofloxacin (CIP) significantly reduced their aerosol performance after 4 months at 25 °C and 20% RH [13]. In another study, L-leucine provided short-term stability to spray-dried CIP DPI formulations at 20 °C and 55% RH [14]. In both studies, the stored formulations demonstrated comparably stable states at low temperatures and RH. Thus, particle-engineered techniques have been applied to develop more stable formulations in adverse conditions including spray-dried particles, drug-polymer conjugates and drug-loaded polymeric NPs [15,16,17]. However, it is still very important to determine the optimized conditions to store the DPI formulations to retain their aerosolization properties. Surprisingly very few works have been done so far to confirm the optimized conditions to store the prepared DPI formulations.

Polymers as carriers protect the drug from degradation thus ensuring stability in adverse conditions including unfavourable temperature and humidity [18]. The polymeric drug carriers could control the formulation’s particle size, shape, and surface properties [1]. Thus, polymers could be an alternative to protect the drug from moisture absorption and maintain the desired flow properties of the formulated DPI formulations. Surprisingly, polymers have rarely been used to stabilize formulated drug molecules against unfavourable temperatures and RH. Natural polymers are now the top picks as nanocarriers because of their biocompatibility, controlled enzymatic degradation, and easily tunable properties [19]. However, the biological safety profile of natural polymers such as chitosan is still to be determined [20]. Thus, the search for suitable polymers for drug delivery is still the focal point of research. Recently, we developed the inhaled CIP-loaded poly(2-ethyl-2-oxazoline) (PEtOx) nanoparticles (NPs) in the form of a DPI formulation [17]. The prepared NPs demonstrated an initial burst release of 50% CIP within the first 12 h and then controlled release for up to 7 days. Additionally, the formulation demonstrated increased aerosolization properties with increased drug loading. The selection of polymer PEtOx was done considering its excellent application in biomedical fields including drug delivery. It is an amorphous polymer that is prepared by the cationic ring-opening polymerization of a 2-ethyl-2-oxazoline monomer. It is a non-ionic polymer attractive for drug delivery because of its unique characteristics including high solubility in water and polar organic solvents, non-toxic behaviour, and high thermal stability [21]. Additionally, PEtOx is known to demonstrate pH-responsive drug release characteristics in biomedical applications [22]. Moreover, PEtOx NPs demonstrated significantly lower non-specific cellular uptakes compared to poly(ethylene glycol) (PEG) NPs with improved stealth modification of the surface properties of the NPs [23]. NPs usually provide benefits of higher surface areas, high solubility, improved biodistribution, and degradation over other carriers such as nanogels [24]. Additionally, the nanocarriers are usually free from surfactants and toxic crosslinking agents due to the repeated washings of the formulated compounds thus reducing the cytotoxic effects [25]. As discussed above, dry powders usually lose their physical stability in inappropriate temperatures and humidity thus adversely affecting the aerosol performance of the formulated compounds. Thus, it is important to determine the storage stability of the blank and CIP-loaded PEtOx NPs to determine their shelf life for clinical applications. To date, the storage stability of CIP-loaded PEtOx NPs has not been studied for inhalation therapy.

Therefore, this study aimed to determine the storage stability of the formulated CIP-loaded PEtOx NPs at different temperatures and humidity. The NPs were prepared using the optimized conditions which were reported in our previous study [17]. In the present study, the storage stability of blank and CIP-loaded PEtOx NPs was studied in 25 °C/43% RH and 40 °C/75% RH. The stored powder samples were investigated to determine the particle sizes and size distribution, morphology, solid-state properties, thermal behaviour, drug-polymer interactions, and aerosolization properties.

## 2. Results and Discussion

### 2.1. Particle Size and Size Distribution

**Figure 1:**
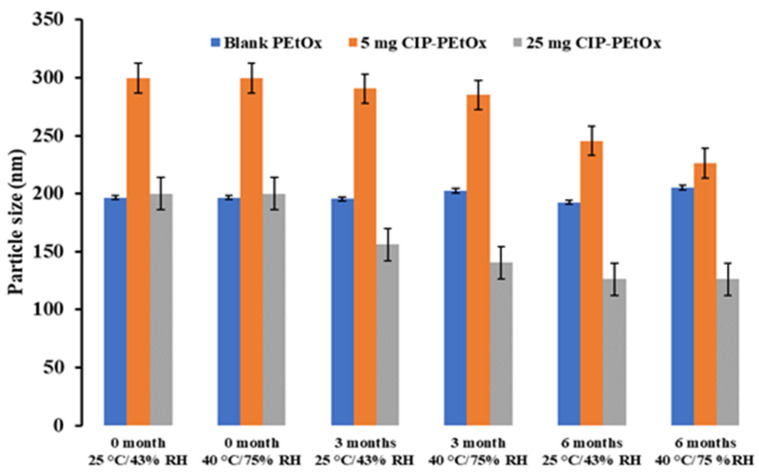
The average particle size of blank and CIP-loaded PEtOx NPs before and after storage at 25 °C/43% RH and 40 °C/75% RH. (data mean with ±SD, *n* = 3).

**Figure 2:**
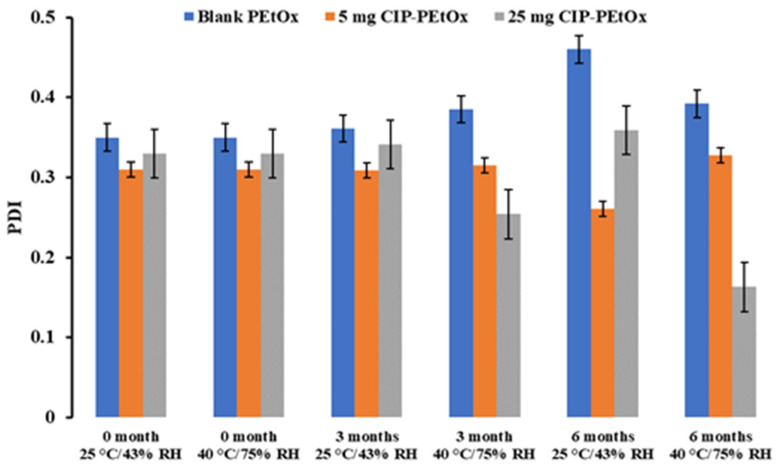
The average particle size distribution or PDI of blank and CIP-loaded PEtOx NPs before and after storage at 25 °C/43% RH and 40 °C/75% RH (data mean with ±SD, *n* = 3).

The average particle size of the original blank PEtOx NPs is about 196.7 ± 24.0 nm and 5 mg and 25 mg CIP-loaded PEtOx NPs are 299.7 ± 18.2 nm and 200.0 ± 22.0 nm, respectively, as reported previously [17]. Stored blank and CIP-loaded PEtOx NPs’ average particle size and size distribution are presented in Figure 1 and Figure 2, respectively, compared with freshly prepared samples.

**Figure 3:**
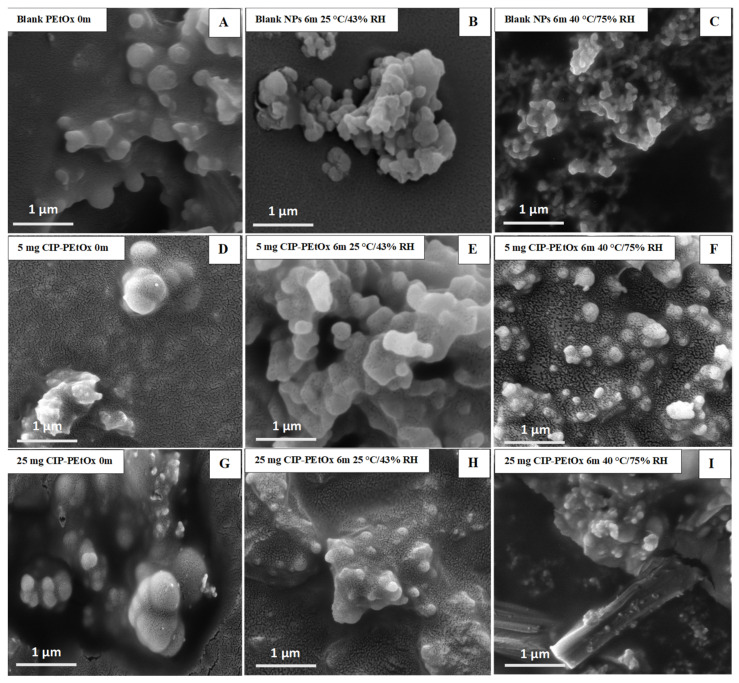
SEM images of blank and CIP-loaded PEtOx NPs before (0 m) and after storage for six months (6 m) at 25 °C/43% RH and 40 °C/75% RH; (**A**–**C**) blank PEtOx NPs; (**D**–**F**) 5 mg CIP-loaded PEtOx NPs; (**G**–**I**) 25 mg CIP-loaded PEtOx NPs.

The average particle size of blank PEtOx NPs stored at 25 °C/43% RH remained close to the initial particle size of 196 nm (*p*-value = 0.2) after 3 and 6 months. However, the particles stored at 40 °C/75% RH showed a significantly increased particle size (*p* < 0.05) due to the moisture absorption and agglomerations among the particles [26]. Similar findings were observed in both conditions with the particle size distributions (Figure 2). However, blank PEtOx NPs showed slightly increased particle size distribution after 6 months compared to 3 months of storage at 40 °C/75% RH. On the other hand, the particle size of 5 mg CIP-loaded PEtOx NPs did not show significant differences (*p* = 0.2–0.3) after 3- and 6-month storage periods under the same conditions. As reported in our previous study, according to the SEM images CIP molecules are present on the surface of the polymeric NPs and might prevent moisture absorption compared to the moisture absorption of blank PEtOx NPs (Figure 3) [17]. Thus, 5 mg CIP-loaded PEtOx NPs did not show a significant increase in particle sizes upon the completion of the storage periods. On the other hand, 25 mg CIP-loaded PEtOx NPs revealed significantly (*p* < 0.05) lower particle size after a 6-month storage period at 40 °C/75% RH conditions. However, these particles maintained close particle sizes to the initial sizes of 200 nm (*p* > 0.05) at 25 °C/43% RH condition. No significant differences were observed among the particle size distributions of 5 mg and 25 mg CIP-loaded PEtOx NPs in any of the conditions (Figure 2). The findings revealed that blank PEtOx NPs maintained the particle size close to its initial particle size at room temperature (25 °C) and relative humidity (43% RH). However, CIP-loaded PEtOx NPs showed reduced particle sizes from 200.2 nm to 126.3 nm after 6 months at high temperatures (40 °C) and RH (75% RH). CIP-loaded PEtOx NPs showed decreased particle sizes because of polymer fusion at high temperatures and drug decay from the surface of the polymer, which matches previous findings [10,13]. According to the scanning electron microscopy (SEM) images and dispersibility study, the smooth surfaces of the CIP-loaded PEtOx NPs and reduced aerosolization also revealed that the drugs might have decayed from the surface of the polymer. The freshly prepared freeze-dried particles demonstrated increased particle sizes after the drying process thus detailed characterization was carried out in our previous study to confirm the chemical stability of the formulated NPs before and after freeze-drying [17]. In this study, the particle size and chemical stability of the NPs were performed to understand the hygroscopic behaviour of PEtOx polymer and its formulations at high temperatures and RH. The increased particle size of the blank PEtOx NPs confirmed the moisture absorption of the polymer at high RH conditions. Thus, PEtOx polymer is not capable to protect the formulation from absorbing moisture at high RH. Therefore, it is anticipated that the aerosolization properties of the prepared DPI formulations might be significantly impacted at high RH.

### 2.2. Moisture Content on Loss-on-Drying

Table 1 represents the moisture content of the formulated NPs. Blank and 5 mg CIP-loaded PEtOx NPs demonstrated constant weight after 6 h of drying. However, 25 mg CIP-loaded PEtOx NPs showed constant weight after 12 h of the drying process. The statistical analysis demonstrated no significant loss of weight occurred during the drying process among the formulations. Blank PEtOx NPs demonstrated the highest weight loss due to the inbound water accumulated during the preparation of the formulation. However, the weight loss was not significant (*p* > 0.05) and thus the moisture content was very low within the formulated NPs despite the hydrophilic nature of the PEtOx polymer. Due to the higher amount of CIP within the formulations, 25 mg CIP-loaded PEtOx NPs showed slow weight loss, which retained the inbound accumulated water during the drug-loading process. However, the low moisture content within the formulated NPs demonstrated the effective drying of the pharmaceutical compounds in a freeze-dryer [27]. The low amount of moisture content within the formulated NPs was not significant and did not affect the aerosolization properties.

### 2.3. Morphology Analysis of Stored Samples by Scanning Electron Microscopy (SEM) and Transmission Electron Microscopy (TEM)

**Figure 4:**
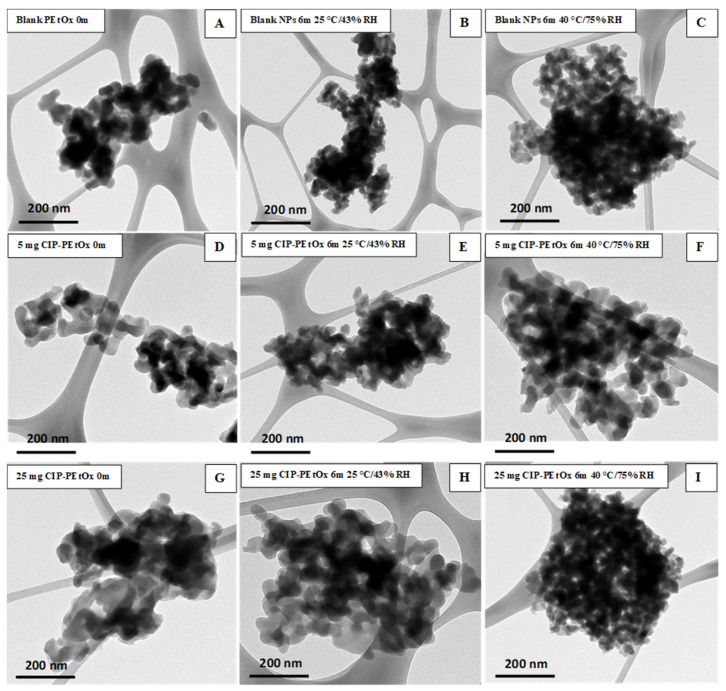
TEM images of blank and CIP-loaded PEtOx NPs before (0 m) and after the storage for six months (6 m) at 25 °C/43% RH and 40 °C/75% RH; (**A**–**C**) blank PEtOx NPs; (**D**–**F**) 5 mg CIP-loaded PEtOx NPs; (**G**–**I**) 25 mg CIP-loaded PEtOx NPs.

The stored samples were studied by SEM and TEM to understand the morphology of the NPs. Blank PEtOx and CIP-loaded PEtOx NPs showed smooth and rough surfaces, respectively, in SEM images, as reported in our previous study [17]. However, all the particles appeared as more agglomerates after the storage periods compared with the freshly prepared NPs as presented in SEM and TEM images (Figure 3 and Figure 4). However, SEM images demonstrated more aggregations among the particles and resulted in larger particle sizes compared to DLS measurement. As DLS measured the liquid suspension of the particles thus the particles were deagglomerated during the sonication and resulting in decreased particle sizes [28]. Similar type of particle aggregations was also observed after storing the NPs (Figure 4). Both the SEM and TEM images confirmed that the powder samples tend to aggregate with the increasing temperatures and RH. Therefore, all the samples demonstrated more agglomerates behaviour at 40 °C/75% RH compared to 25 °C/43% RH. Although blank PEtOx NPs showed similar surface properties (Figure 3A–C), CIP-loaded PEtOx NPs showed noticeable changes after the completion of the storage periods (Figure 3D–I). CIP-loaded PEtOx NPs of 5 mg and 25 mg demonstrated smooth surfaces compared to those of freshly prepared particles (rough surfaces) with reduced particle sizes (Figure 3D–I). Thus, the reduced particle sizes of CIP-loaded PEtOx NPs match with the particle size analysis in DLS measurement. The reduced particle sizes and more smooth surfaces of CIP-loaded PEtOx NPs might occur due to the drug decay from the polymeric surface at high temperature [10]. The surface smoothness was not comparable with the freshly prepared samples and stored samples as evidenced by the TEM images. Therefore, TEM images could not provide much information on the drug decay from the polymeric surface. However, the increased aggregation at high temperature and RH caused to increase the particle size, which matched the findings of SEM and DLS studies. Although high temperatures could also fuse the surface of the polymeric NPs it was not visible in the SEM and TEM images. This may have contributed to the reduced FPF compared to freshly prepared NPs.

### 2.4. Powder X-ray Diffraction: Solid-State

**Figure 5:**
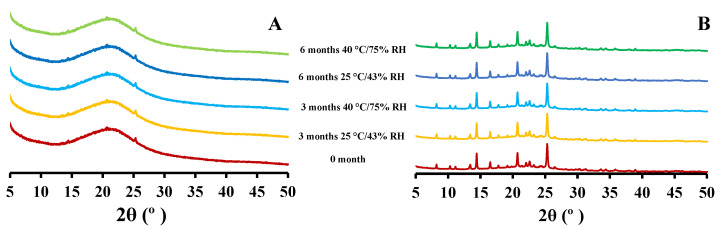
X-ray powder diffraction patterns of CIP-loaded PEtOx NPs before and after 3 and 6 months of storage at 25 °C/43% RH and 40 °C/75% RH; (**A**) 5 mg CIP-loaded PEtOx NPs; (**B**) 25 mg CIP-loaded PEtOx NPs.

The X-ray diffraction of 5 mg and 25 mg CIP-loaded PEtOx NPs showed similar patterns before and after storage periods (Figure 5) as reported in your previous study [17]. The changed surface morphology (according to SEM images) of CIP-loaded PEtOx NPs after 6 months of storage conditions was not reflected in the PXRD data. The crystalline CIP powder did not reveal any new intensity peaks or changed intensity peaks. Thus, this indicates that the crystallinity of the formulated CIP remained unchanged after the storage periods [13]. X-ray diffraction measurement cannot detect less than 10% crystallinity differences among the samples [29,30]. Thus, the findings suggest that the crystallinity differences among the stored CIP-loaded PEtOx NPs were between 1–10%. The findings also revealed that there were no chemical interactions between the drug and the polymer during the storage periods.

### 2.5. Thermal Analysis

**Figure 6:**
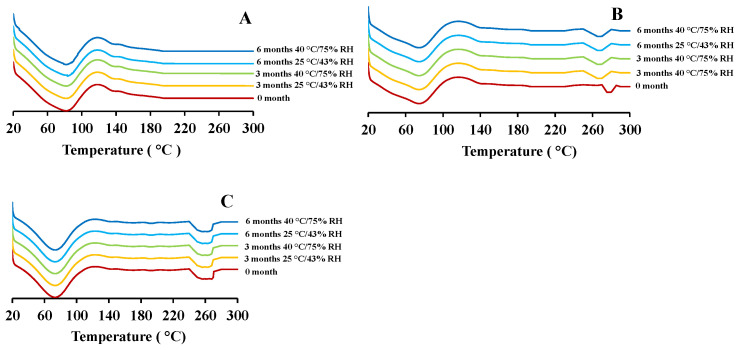
DSC thermograms of blank and CIP-loaded PEtOx NPs before and after 3 and 6 months of storage at 25 °C/43% RH and 40 °C/75% RH; (**A**) blank PEtOx NPs; (**B**) 5 mg CIP-loaded PEtOx NPs; (**C**) 25 mg CIP-loaded PEtOx NPs.

As reported in our previous study, the freshly prepared blank PEtOx NPs demonstrated a broad endothermic peak at 65.1 °C and the maximum peak was observed at 82.9 °C (Figure 6A) [17]. After storage, the NPs demonstrated similar endothermic peaks except for the samples stored for 6 months at 40 °C/75% RH condition. The endothermic peak was shifted to 89.1 °C due to the moisture absorption at high RH and increased glass transition temperature [13]. CIP-loaded PEtOx NPs demonstrated two broad endothermic peaks before the storage periods, one was comparable with the peaks of blank PEtOx NPs and another one was comparable with the melting temperature of CIP drug [17]. After storage periods, all the samples demonstrated similar endothermic peaks as shown in the first endothermic peaks before the storage periods. However, the second endothermic peaks were found to shift slightly in lower points (240–270 °C) as compared with the samples before the storage periods (Figure 6B,C). The shifted endothermic peaks were attributed to the dehydration of the CIP accumulated during the storage periods at high RH conditions [14]. However, the shift did not indicate the changed crystalline nature of the drug molecules as it was not detectable in X-ray diffraction patterns. The shift might occur due to the absorption of water at high relative humidity and dehydration during the DSC test [10]. In a previous study, nicotine-loaded chitosan NPs retained the same physical characteristics after one-year storage at 25 °C/60% RH because of the hydrophobic nature of the chitosan polymer [31]. However, PEtOx is hydrophilic and might absorb a significant amount of moisture at high RH. Therefore, the hygroscopic nature of the formulations could significantly decrease the aerosolization properties.

### 2.6. Attenuated Total Refection-Fourier Transform Infrared (ATR-FTIR)

**Figure 7:**
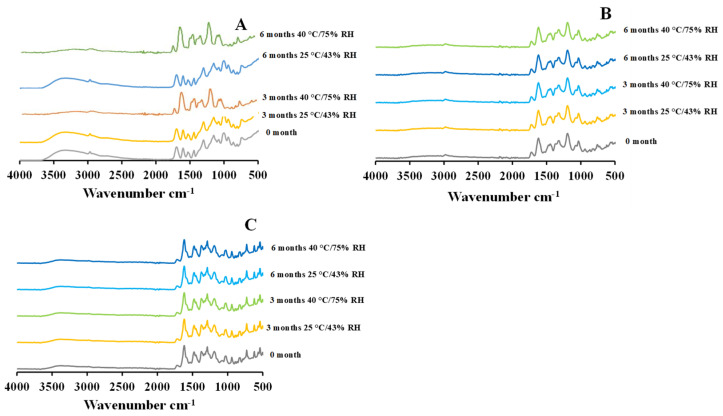
FTIR spectrum of blank and CIP-loaded PEtOx NPs before and after 3 and 6 months of storage at 25 °C/43% RH and 40 °C/75% RH; (**A**) blank PEtOx NPs; (**B**) 5 mg CIP-loaded PEtOx NPs; (**C**) 25 mg CIP-loaded PEtOx NPs.

The FTIR spectra of 5 mg and 25 mg CIP-loaded PEtOx NPs demonstrated similar characteristic peaks before and after storage periods (Figure 7B,C) as reported in your previous study [17]. The stored CIP-loaded PEtOx NPs showed their characteristic peaks of N-H bending vibration of quinolines at 1616 cm^−1^ and C=O stretching vibration of the carbonyl group at 1718 cm^−1^ in all the storage conditions which match exactly with the characteristic peaks of the samples before storage (Figure 7B,C). This confirms there was no chemical reaction between CIP and the polymer during the storage periods [32]. The presence of the C=O stretching vibration of the carbonyl group also suggests the presence of hydrogen bonding after the storage periods and maintains the particle formation characteristics as evidenced by the freshly prepared samples [17]. The findings revealed that CIP-loaded PEtOx NPs maintain their chemical stability during the preparation and storage periods [33]. On the other hand, blank PEtOx NPs at 25 °C/43% RH demonstrated similar characteristic peaks of the hydrogen bonding between the polymer and the tannic acid at 1603 cm^−1^, as reported in our previous study [17]. However, the characteristic peak of blank PEtOx NPs stored at 40 °C/75% RH disappeared from 2927 cm^−1^ (Figure 7A). This might occur due to the breaking of the N-H bending vibration of the blank PEtOx NPs at high temperatures and relative humidity. Thus, some molecules of the formulated NPs might have returned to their original form of polymorphism during the storage period [34]. However, as both the powders of CIP-loaded PEtOx NPs maintained their chemical stability after the completion of storage periods, the CIP-loaded PEtOx NPs are chemically stable at high temperatures and relative humidity, which also matches our PXRD findings.

### 2.7. In Vitro Aerosol Performance

**Figure 8:**
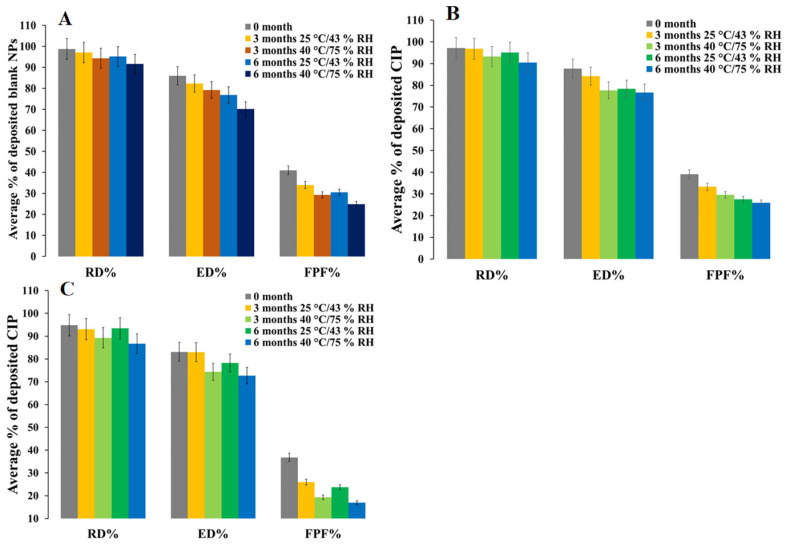
In vitro aerosol performance of blank and CIP-loaded PEtOx NPs before and after 3 and 6 months of storage at 25 °C/43% RH and 40 °C/75% RH; (**A**) blank PEtOx NPs; (**B**) 5 mg CIP-loaded PEtOx NPs; (**C**) 25 mg CIP-loaded PEtOx NPs.

An in vitro aerosol performance study reveals 41.0%, 36.8%, and 39.1% fine particle fraction (FPF) for freshly prepared blank PEtOx, 5 mg, and 25 mg CIP-loaded PEtOx NPs, respectively (Figure 8). Blank PEtOx NPs showed better dispersibility behaviour compared to CIP-loaded PEtOx NPs. However, all the formulations showed significantly lower (*p* < 0.05) FPF after 3- and 6-month storage periods at 25 °C/43% RH and 40 °C/75% RH. The FPF of blank PEtOx NPs initially dropped 8% (*p* < 0.05) after 3 months, and then it dropped by 10% (*p* < 0.05) after 6 months at 25 °C/43% RH. The reduction in FPF occurred due to the moisture absorption under high humid conditions and increased particle sizes due to the formation of large agglomerates evidenced by the SEM and TEM images [35]. In the storage conditions especially in the high RH, the formed agglomerates had lower flow properties compared to the freshly prepared NPs and resulting in reducing the FPF (Figure 3A). Although there was no significant difference (*p* > 0.05) between the FPF of 3-month and 6-month storage periods at 25 °C/43% RH, significant differences (*p* < 0.05) were observed between the FPFs of 3-month and 6-month storage periods at 40 °C/75% RH. This confirms more moisture absorption in high humid conditions compared to that of low humid conditions and results in reduced FPF. On the other hand, the FPF dropped between 6–20% in the case of CIP-loaded PEtOx NPs. Significantly reduced (*p* < 0.05) FPFs were observed with CIP-loaded PEtOx NPs in all conditions in comparison with the freshly prepared CIP-loaded PEtOx NPs (Figure 8B,C, Table 2). The reduced FPFs in the case of CIP-loaded PEtOx NPs occurred due to the drug decay from the polymeric surface of the NPs as evidenced in the SEM images (Figure 3D–I) [10]. In addition, inter-particular capillary forces among the particles might also increase due to the significant amounts of moisture absorption under humid conditions and resulting in reduced FPF [36,37]. Although polymer fusion at high temperatures could be another reason for drug decay from the surface of the polymeric NPs and reduced FPF, it was not visible in the SEM and TEM images (Figure 3 and Figure 4). There was no significant difference among the RD of the formulated NPs throughout the storage periods. However, the ED of blank PEtOx NPs dropped significantly (*p* < 0.05) after the storage periods under both conditions. Similar findings were also observed in the case of CIP-loaded PEtOx NPs except for 3-month storage periods at 25 °C/43% RH (*p* > 0.05). The FPD of 5 mg CIP-loaded PEtOx NPs declined from 0.7 to 0.5 mg (*p* > 0.05) after 6 months at 25 °C/43% RH condition (Table 2). However, the FPD reduced significantly (*p* < 0.05) after 6 months at 40 °C/75% RH. On the other hand, the FPD of 25 mg CIP-loaded PEtOx NPs declined from 4.5 to 3.9 mg (*p*-value = 0.035679) after a 3-month storage period at 25 °C/43% RH condition. However, all other experiments revealed significantly (*p* < 0.05) lower FPD in both the conditions after 3-month and 6-month storage periods. Similar findings were also observed in a study when CIP in combination with phage PEV20 was stored at high relative humidity for 4 months [13]. Thus, the reduced aerosolization properties of the formulated NPs indicate that they are not physically stable at high temperatures and RH for a long period, thus the formulations should be stored at low temperatures and RH.

## 3. Materials and Methods

### 3.1. Materials

HPLC grade CIP powder MW 331.34 g/mol (assay: ≥98.0%); PEtOx MW 50 kDa; tannic acid (TA) MW 1701.20 g/mol; sodium chloride (NaCl) anhydrous (ACS reagent, ≥99%) were purchased from Sigma-Aldrich Pty Ltd. (Castle Hill, NSW, Australia). Potassium carbonate anhydrous (Potash) was purchased from ChemSupply Australia (Gillman, SA, Australia). All other reagents and solvents were used of analytical grade.

### 3.2. Preparation of Blank PEtOx NPs

The blank PEtOx NPs were prepared using the previous study conducted by Sabuj et al. [17]. In brief, PEtOx polymer was dissolved in deionized water (1% *w*/*v*) and TA was separately dissolved in the same amount of deionized water (0.03% *w*/*v*). Then, the TA solution was mixed with the PEtOx solution dropwise over a magnetic stirrer to form NPs in a coassembly reaction. The addition of the TA solution made the mixture turbid and confirmed the formation of the NPs. Then, the NPs were separated by centrifugation and freeze-dried using an Alpha 1-4 LD plus Freeze Dryer.

### 3.3. Preparation of CIP-Loaded PEtOx NPs

From our previous study [17] two batches of CIP-loaded PEtOx NPs were selected to examine whether the amount of CIP within the formulated NPs affects the storage stability. The methods of preparing CIP-loaded PEtOx NPs were similar to the blank PEtOx NPs preparation except for the addition of CIP within the formulations. In brief, one batch of CIP-loaded PEtOx NPs was prepared by dissolving 0.025% CIP into a TA solution (0.03% *w*/*v*) and the second batch was prepared by dissolving 0.125% CIP into another TA solution (0.06% *w*/*v*). Then, the mixtures were separately added dropwise with the PEtOx solution to form the CIP-loaded PEtOx NPs. Each batch of the formulated NPs produced 5 mg and 25 mg CIP in weights within each formulation, respectively. The compositions of these two batches with the blank formulation are summarized in Table 3.

### 3.4. Determination of Moisture Content

The freshly prepared freeze-dried NPs were investigated for their moisture content in a modified loss-on-drying method [38]. Briefly, 500 mg of blank PEtOx, 5 mg CIP-loaded PEtOx, and 25 mg CIP-loaded PEtOx NPs were stored separately in an oven at 40 °C. This gravimetric method was carried out using a weighing boat. The weight of the preweighed weighing boat was validated by drying the boat at 40 °C until reaching a constant weight. Three blank weighing boats were used in three replicates to validate this method and an average constant weight of the boat was used to determine the weight of the experimental samples. The samples were weighed after 1 h, 2 h, 6 h, 12 h, and 24 h to reach a constant weight of the NPs. The weight of the oven-dried NPs was determined by subtracting the weight of the blank weighing boat from the total weight.

### 3.5. Stability Assessment

Blank PEtOx, 5 mg, and 25 mg CIP-loaded PEtOx NPs were examined for a long-term stability study under two controlled conditions of 25 °C/43% relative humidity (RH) (normal environmental condition) and 40 °C/75% RH. All the powder samples were kept in stability chambers under the specified conditions for durations of 3 and 6 months. Acceptable RH of 43% and 75% were maintained by saturated potassium carbonate and sodium chloride solutions, respectively [39]. These two conditions (25 °C/43% RH and 40 °C/75% RH) were selected to determine the stability and the effects of room temperature and normal RH and high temperature and RH on the aerosol performance of the formulated NPs. The physiological characteristics of the NPs were assessed after 0, 3, and 6 months of storage.

### 3.6. Morphology Investigation

The surface morphology of the powder samples was determined by scanning electron microscopy (SEM) (Tescan Mira3 Brno, Czech Republic) and transmission electron microscopy (TEM) (JOEL 1400 TEM). Stored blank and CIP-loaded PEtOx powder samples were analysed in SEM at 5 kV stepping-up voltage. Before that, the powder samples were scattered on a carbon adhesive tape attached to the surface of an aluminium stub. Finally, all the samples were coated with a 5 nm thick gold coater. Analysis was done using the SEM working distance of 8.08 mm.

Using a TEM, the powder samples were further analysed to understand the stability of NPs at highly stressed conditions. The powder samples were mounted on a 300 mesh copper grids coated with lacey carbon. The copper grids were dipped onto sample container lip to pick up a trace amount of fine powder sample. Then, the grids were laid on parafilm and coated with a 5 nm-thick carbon coater. Analysis was carried out at the accelerating voltage of 80 kV.

### 3.7. Powder X-ray Diffraction (PXRD)

The crystallinity of the CIP-loaded PEtOx NPs was carried out by powder X-ray diffraction (Rigaku^®^, Tokyo, Japan) instrument. The instrument operating parameters were used as follows: 40 kV Cu-Kα radiation, 40 mA current, 2θ range of 3–120°, and a scan speed of 2.0°/min.

### 3.8. Thermal Analysis

Thermal stability of the stored blank PEtOx and CIP-loaded PEtOx NPs were examined by a differential scanning calorimetric (DSC) instrument (TA Instruments DSC; model Q100). Powder samples (2 ± 0.1 mg) were weighed accurately and mounted into an aluminium crucible. An empty customized aluminium crucible was used as a reference. All the crucibles were sealed with a perforated lid. Finally, the samples were scanned under a nitrogen atmosphere from the temperature range of 20–300 °C. The heating rate was set at 5 °C/min. Samples were analysed in triplicate.

### 3.9. Attenuated-Total Refection-Fourier Transform Infrared (ATR-FTIR)

Stored blank and CIP-loaded PEtOx powder samples were examined by a Thermo iS50 FTIR spectrometer (Nicolet, Madison, WI, USA) to obtain their ATR-FTIR spectra. The instrument was equipped with a single reflection diamond crystal with an angle of incidence of 40° and a deuterated triglycine sulphate (DTGS) detector. A small amount of powder sample was secured on top of the diamond crystal with a high-pressure clamp. Instrument parameters were selected as follows: the resolution of 8 cm^−1^, number of scans 64, range of data collection 4000–400 cm^−1^. The collected data were analysed using the OMNIC analytical software (Nicolet Instrument Corp., Version 9.2, Madison, WI, USA).

### 3.10. In Vitro Aerosol Performance of the Stored Samples

The aerosolization study of the stored blank PEtOx and CIP-loaded PEtOx NPs was performed using a twin-stage impinger (TSI, British Pharmacopoeia, 2020) following a previously reported method [17]. In brief, formulated particles were inserted into size 3 hard gelatine capsules (Fawns and McAllan Pty., Belmont, Australia). Each capsule had 20 ± 1 mg powder samples for the aerosolization study. Then, the capsules were inserted into a device, Breezehaler^®^ (Novartis Pharmaceuticals Pvt Ltd., Macquarie Park, NSW, Australia). The TSI had another two parts which are known as Stage 1 (S1) and Stage 2 (S2). During the experimentation 7 mL and 30 mL PBS (pH 7.4 ± 2) (as solvent) were dispensed in S1 and S2 of the TSI device, respectively. After securing all the parts together a controlled airflow (60 ± 5 L/min) was drawn for 5 s to disperse the powder particles from the Breezehaler^®^ using a vacuum pump (D-63150, Erweka, Germany). A digital calibrated flow meter (Fisher and Porter, Model 10A3567SAX, UK) was used to control the airflow rate through the vacuum pump. After each experimentation, the aerosolized particles were collected by washing all three parts of the TSI separately. Before further experimentations, the collected samples were kept in optimized conditions (37 °C and 100 rpm over a magnetic stirrer) for 7 days to release CIP from the CIP-loaded PEtOx NPs [17].

The CIP released samples were analysed both in gravimetric and quantitative methods. The gravimetric analysis was carried out by repeated filtration of CIP-released NPs using a pre-dried and pre-weighed filter paper (orifice 0.20 µm, Phenomenex, Torrance, CA, USA) until the solutions showed any sign of turbidity. Then, the filter papers were dried in an oven at 60 °C for 24 h to obtain a constant weight of the accumulated particles on the surface of the filter paper. Then, the filtrated solutions were studied in the high-performance liquid chromatography (HPLC) method to determine the concentration of released CIP from the CIP-loaded PEtOx NPs [17]. Then, the findings from gravimetrical and analytical methods were added together to determine the final concentration of CIP dispersion in each stage of the TSI device. However, blank CIP-loaded PEtOx NPs were only analysed using the gravimetrical method.

The dispersibility of the stored blank PEtOx and CIP-loaded PEtOx NPs was determined in four parameters, including, recovered dose (RD), emitted dose (ED), fine particle fraction (FPF), and fine particle dose (FPD). RD was calculated as the percentage of the total amount of drug deposited into Breezhaler^®^, S1, and S2 of the TSI device. ED was determined by calculating the percentage of the emitted drug into S1 and S2. FPF was calculated in percentage to determine the respirable fraction of the stored blank PEtOx and CIP-loaded PEtOx NPs. FPD is the amount of drug deposited into the S2 of the TSI device. Equations (1) and (2) were used to calculate these parameters.

(1) $$\mathrm{ED}=\frac{S1+S2}{\mathrm{RD}}\times 100$$

(2) $$\mathrm{FPF}=\frac{S2}{\mathrm{RD}}\times 100$$

### 3.11. Quantitative Analysis of CIP by High-Performance Liquid Chromatography (HPLC) Method

A previously published validated method [17] was used to determine the concentration of CIP by using an Agilent HPLC Series 1100 (Waldbronn, Germany) instrument consisting of an autosampler and diode array detector (Atlas) Hewlett-Packard. The instrument was operated by the online ChemStation software. The applied parameters used in this experiment were as follows: Agilent Poroshell 120 EC-C18 (4 µm, 4.6 × 250 mm) column (USA) as stationary phase, a mixture of 0.1% trifluoracetic acid in water (pH 2.0), and 100% acetonitrile (80:20 *v*/*v*) as the mobile phase at a flow rate of 1 mL/min, 10 µL injection volume, total run time 10 min, UV absorbance detector at 275 nm wavelength for CIP quantification. A standard linear curve (R^2^ = 0.924) was built by dissolving CIP in PBS over the concentration range of 1.0–100 µg/mL with the limit of quantification of 1 µg/mL.

### 3.12. Statistical Analysis

The findings were calculated as mean values and standard deviations from three measurements. Statistical analysis was performed in one-way analysis of variance (ANOVA) between the groups. Probability (*p*) values of <0.05 were considered statistically significant differences.

## 4. Conclusions

In this study, the stability of CIP-loaded PEtOx NPs was examined at normal room conditions (25 °C/43% RH) and high temperature and RH (40 °C/75% RH). Blank PEtOx NPs and CIP-loaded PEtOx NPs remained chemically stable under both conditions. However, the physical characteristics of the formulated NPs were compromised significantly throughout the storage periods. The aerosol performance of the formulated NPs reduced significantly at high temperatures and RHs. All the formulations formed agglomerations during the storage periods and CIP-loaded PEtOx NPs demonstrated a significantly smooth surface as compared to the freshly prepared formulations. The smooth surface properties of the drug-loaded PEtOx NPs confirmed by SEM images indicate a significant amount of CIP decay occurred from the polymeric surface at high temperatures and RH. Thus, the PEtOx polymer failed to retain the aerosolization properties of the formulated CIP-loaded PEtOx NPs for six months under storage conditions. The outcome of this study revealed that the prepared CIP-loaded PEtOx NPs are not stable at high temperatures and RH. Therefore, further study is warranted to determine the optimized conditions to store the formulated NPs and determine their shelf-life.

## Figures and Tables

**Table 1:** Weights of the compounds in the loss-on-drying process (data mean with ± S.D., *n* = 3).

Formulated NPs	Weight after 1 h	Weight after 2 h	Weight after 6 h	Weight after 12 h	Weight after 24 h
Blank PEtOx NPs	492.2 ± 0.2	492.1 ± 0.1	492 ± 0.1	492 ± 0.1	492 ± 0.1
5 mg CIP-loaded NPs	498.1 ± 0.3	498.1 ± 0.2	496.8 ± 0.4	496.7 ± 0.2	496.7 ± 0.1
25 mg CIP-loaded NPs	498.4 ± 0.7	496.8 ± 0.8	495.8 ± 0.5	494.7 ± 1.7	494.7 ± 0.4

**Table 2:** Aerosol performance of blank and CIP-loaded PEtOx NPs after 3 months and 6 months of storage at 25 °C/43% RH and 40 °C/75% RH.

Formulations	Storage Conditions	Storage Period	FPF (%)	FPD (mg)
Blank PEtOx NPs	25 °C/43% RH	3	32.3 ± 0.8	-
		6	30.5 ± 2.4	-
	40 °C/75% RH	3	29.3 ± 0.7	-
		6	24.9 ± 2.0	-
5 mg CIP-loaded NPs	25 °C/43% RH	3	25.9 ± 1.9	0.6 ± 0.1
		6	20.7 ± 2.1	0.5 ± 0.2
	40 °C/75% RH	3	19.3 ± 1.0	0.44 ± 1.1
		6	16.9 ± 3.1	0.4 ± 0.3
25 mg CIP-loaded NPs	25 °C/43% RH	3	33.3 ± 0.6	3.9 ± 0.6
		6	27.5 ± 1.1	3.6 ± 0.1
	40 °C/75% RH	3	29.7 ± 1.5	3.4 ± 0.4
		6	25.9 ± 0.5	3.1 ± 0.1

**Table 3:** Compositions of the compounds used in this study to formulate the NPs.

Formulated NPs	Amount of PEtOx (mg)	Amount of TA (mg)	Amount of CIP (mg)
Blank PEtOx NPs	200	6	-
5 mg CIP-loaded NPs	200	6	5
25 mg CIP-loaded NPs	200	12	25

## Data Availability

Data is contained within the article.
